# Robot-assisted training for post-stroke lower-limb dysfunction: an umbrella review

**DOI:** 10.3389/fpubh.2026.1969488

**Published:** 2026-10-16

**Authors:** Lei Wang, Can Wang, Qian Cao, Keping Yin, Lingnan Ding, Ailian Chen

**Affiliations:** 1 Hunan Provincial People’s Hospital and The First-Affiliated Hospital of Hunan Normal University, Changsha, China; 2 College of Medicine, Hunan Normal University, Changsha, China

**Keywords:** lower limb motor function, rehabilitation, robot, stroke, systematic review, umbrella review

## Abstract

**Objective:**

This study aimed to reappraise published systematic reviews and meta-analyses on robot-assisted training for lower-limb motor dysfunction after stroke and evaluate their methodological quality, reporting completeness, and certainty of evidence.

**Methods:**

Seven databases were searched, including CNKI, Wanfang, VIP, PubMed, Cochrane Library, Embase, and Web of Science, from inception to January 15, 2026. Eligible studies were systematic reviews or meta-analyses that were based on randomized controlled trials. Two reviewers independently performed study selection, data extraction, and quality appraisal. The methodological quality of the included reviews was assessed using A MeaSurement Tool to Assess systematic Reviews (AMSTAR) 2, and the reporting of this umbrella review followed the Preferred Reporting Items for Overviews of Reviews (PRIOR) guideline. The reporting completeness of the included systematic reviews and meta-analyses was evaluated using the Preferred Reporting Items for Systematic Reviews and Meta-Analyses (PRISMA) 2020 checklist. The certainty of evidence was graded using the Grading of Recommendations Assessment, Development and Evaluation (GRADE) framework.

**Results:**

Nineteen reviews were included, comprising eight Chinese and 11 English publications. Primary-study overlap across reviews was moderate (CCA = 7.44%). According to AMSTAR 2, three reviews were rated as high quality, six as moderate quality, six as low quality, and four as critically low quality. PRISMA completeness scores ranged from 14.5 to 23.5 out of a maximum possible score of 27. Among the 70 extracted outcomes, none was rated as high-certainty evidence, 20 were of moderate certainty, 32 were of low certainty, and 18 were of very low certainty. Overall, robot-assisted training showed potential benefits for balance and lower-extremity motor function, whereas evidence for walking-related outcomes remained less consistent.

**Conclusion:**

Evidence was most consistent for balance and lower-extremity motor impairment, whereas findings for walking independence, endurance, functional mobility, and gait speed were less consistent and generally supported by low-to-moderate certainty evidence. More rigorously designed primary trials and better-conducted systematic reviews are needed.

**Systematic review registration:**

The review protocol was registered under Prospero, https://www.crd.york.ac.uk/PROSPERO/home (Registration No: CRD420261419692).

## 1 Introduction

Stroke remains the leading cause of death and the third leading cause of death and disability worldwide among noncommunicable diseases (1). Approximately 795,000 individuals experience a new or recurrent stroke annually (2). Over the past several decades, global stroke-related mortality has declined substantially, attributable to advances in diagnostic methodologies, acute interventions, and secondary prevention strategies (3). However, a substantial proportion of stroke survivors develop persistent lower-limb motor impairments, leading to compromised mobility and reduced independence in activities of daily living (ADLs) (4). This not only substantially impairs the quality of life of stroke survivors but also imposes a considerable socioeconomic burden on families and healthcare systems. Consequently, restoring and enhancing lower-limb motor function and ambulatory capacity constitutes a primary clinical objective in post-stroke rehabilitation and represents a key therapeutic target in neurological rehabilitation research and practice (5). Rehabilitation robotics can enhance lower-limb motor function, postural control, and ADLs in individuals with post-stroke hemiparesis, primarily through adjustable partial weight-bearing support and high-repetition, physiologically informed gait training (6). As an emerging modality within contemporary rehabilitation medicine, robotic-assisted gait training has gained increasing clinical adoption globally, and it is now integrated into multidisciplinary rehabilitation programs for diverse neurological and musculoskeletal conditions.

Stroke remains predominantly a disease of older adults, in whom comorbidity, frailty, and pre-existing disability can complicate rehabilitation and increase the importance of preserving mobility and functional autonomy (7). Walking limitations after stroke affect not only performance on impairment-based scales but also independence in activities of daily living, participation, caregiver needs, and long-term care planning. At the population level, the World Stroke Organization-Lancet Neurology Commission estimated the global economic burden of stroke at US$891 billion in 2017, with further growth expected as the number of people living with stroke increases (4). These considerations make it clinically relevant to judge robot-assisted rehabilitation not only by short-term motor outcomes, but also by whether functional gains are meaningful, sustainable, accessible, and feasible across healthcare settings.

In recent years, a number of systematic reviews and meta-analyses of robot-assisted lower-limb functional rehabilitation in patients with stroke have been published, and they have verified the efficacy from the perspective of intervention measures and rehabilitation stages. However, the reliability of its evidence is limited due to inconsistent methodological quality, inconsistent outcome indicators, and non-standard reporting, which is difficult to support clinical decision-making, and may even be misleading. An umbrella review is therefore appropriate not because it can correct weaknesses in the included reviews or upgrade the certainty of their underlying evidence, but because it can bring multiple reviews into a common framework, compare the consistency of their conclusions, critically appraise their methodological and reporting quality, and identify which findings are relatively robust and which remain uncertain. In this sense, the umbrella review provides a higher-order synthesis of the available evidence.

Accordingly, this study systematically reappraised existing systematic reviews and meta-analyses of robot-assisted training for post-stroke lower-limb motor dysfunction. The methodological quality of the included reviews was assessed using AMSTAR 2, their reporting completeness was evaluated using the PRISMA 2020 checklist, and the certainty of outcome-specific evidence was graded using the GRADE framework. The present umbrella review was reported in accordance with the Preferred Reporting Items for Overviews of Reviews (PRIOR) guideline (8). By integrating these complementary assessments, the study aimed to clarify the consistency, reliability, and limitations of the current evidence base and to support appropriately cautious clinical interpretation and future research planning.

## 2 Materials and methods

### 2.1 Study design

This umbrella review systematically re-evaluated existing systematic reviews and meta-analyses on rehabilitation robotics for post-stroke lower-limb motor dysfunction. The design, reporting structure, and presentation of the review were guided by the Preferred Reporting Items for Overviews of Reviews (PRIOR) guideline (8). PRIOR was used as the reporting framework for the present umbrella review, whereas AMSTAR 2, PRISMA 2020, and GRADE were used to appraise the methodological quality, reporting completeness, and certainty of evidence, respectively. The review protocol was registered under Prospero (Registration No: CRD420261419692). All relevant Chinese- and English-language reviews based on randomized controlled trials (RCTs) were included.

### 2.2 Inclusion and exclusion criteria

The literature screening process adhered strictly to the PICOS framework to define eligibility criteria as follows:

(1) Population: adults diagnosed with stroke and presenting with lower limb motor impairment;
(2) Intervention (experimental group): robot-assisted rehabilitation training, either as a standalone intervention or as an adjunct to conventional rehabilitation;
(3) Comparison (control group): conventional (i.e., non-robotic) rehabilitation training;
(4) Outcomes: including the Berg Balance Scale (BBS), Fugl–Meyer Assessment (FMA), Functional Ambulation Category (FAC), Timed Up and Go Test (TUG), 6-Minute Walk Test (6MWT), and 10-Meter Walk Test (10MWT);
(5) Study design: full-text systematic reviews, with or without meta-analysis, that synthesized randomized controlled trials and reported a structured database search, predefined eligibility criteria, explicit study-selection methods, and a reproducible qualitative or quantitative synthesis.

The exclusion criteria included (1) duplicate publications; (2) studies for which the full text was unavailable or that lacked essential methodological or outcome data; (3) non-human experimental studies, conference abstracts and theses/dissertations; (4) publications unrelated to the scope of the present research topic; and (5) Narrative reviews, scoping reviews, nonsystematic reviews, and articles that did not describe a systematic evidence-synthesis process were not eligible.

### 2.3 Search strategy

A systematic literature search was conducted across seven electronic databases, namely, CNKI, Wanfang Data, VIP Chinese Science and Technology Periodical Database, PubMed, the Cochrane Library, Embase, and Web of Science, covering publications indexed up to January 15, 2026. The search strategy employed a combination of controlled vocabulary (e.g., MeSH terms and subject headings in Chinese databases). The English search term is as follows: “robotics/robotic/robot/exoskeleton” AND “cerebrovascular disorders/stroke/cerebrovascular event/poststroke/apoplexy/cerebrovascular accident/brain vascular accident/CVD/cerebral infarction/brain infarction/brain ischemia/intracranial embolism/cerebral hemorrhage/cerebral haemorrhage/brain hemorrhage/brain haemorrhage/intracranial hemorrhage/intracranial haemorrhage,” “Lower limb motor function/lower extremity/walking/walk/balance/gait/lower limb,” and “meta-analysis/systematic reviews.” The full search strategy can be found in Annex 1.

### 2.4 Literature screening and data extraction

Two trained researchers (L W and C W) independently conducted the literature search, screening, and data extraction. EndNote was used to manage references and remove duplicates. Titles and abstracts were screened against the predefined inclusion and exclusion criteria. Full-text articles meeting these criteria were retrieved and independently assessed by both reviewers. Disagreements were resolved through discussion or, if needed, adjudicated by a third senior investigator (AL C). Data extracted included author, publication year, number of studies, intervention protocols (experimental and control groups), and primary and secondary outcomes. All data were entered into Excel and cross-checked by both reviewers; unresolved discrepancies were resolved by the third investigator.

### 2.5 Evaluation methods

#### 2.5.1 Assessment of methodological quality

Two independent reviewers (L W and C W) assessed the methodological quality of the included systematic reviews by using the A MeaSurement Tool to Assess systematic Reviews (AMSTAR) 2 instrument (9). Disagreements between reviewers were resolved through consensus discussion, with adjudication by a third senior reviewer (AL C) when necessary. AMSTAR 2 comprises 16 criteria, seven of which (items 2, 4, 7, 9, 11, 13, and 15) are designated as “critical” items. Per the official AMSTAR 2 scoring guidance, the overall methodological quality of each systematic review was categorized into one of four levels (1): high: no critical item deficiencies and one non-critical item deficiency at most; (2) moderate: no critical item deficiencies but more than one non-critical item deficiency; (3) low: exactly one critical item deficiency (regardless of non-critical item deficiencies); and (4) critically low: two or more critical item deficiencies.

#### 2.5.2 Assessment of reporting completeness

Two independent reviewers (L W and C W) assessed the reporting completeness of each included study by using the PRISMA checklist (10). Discrepancies between reviewers were resolved through consensus discussion, with adjudication by a third senior reviewer (AL C) when necessary. The PRISMA 2020 checklist was used descriptively to assess reporting completeness of 27 reporting items. Item-level adherence was summarized as the proportion of reviews fully or partially reporting each item. No validated methodological-quality classification was inferred from the total PRISMA score.

#### 2.5.3 Evaluation of evidence quality

Two researchers (L W and C W) independently assessed the quality of evidence using the GRADE approach (11). Disagreements were resolved through consensus discussion; when consensus could not be reached, a third senior reviewer (AL C) arbitrated the final judgment. The assessment encompassed five key domains: risk of bias (study limitations), inconsistency of results, indirectness of evidence, imprecision of effect estimates, and potential publication bias. Based on these considerations, the overall certainty of evidence was categorized into four levels: high, moderate, low, and very low.

#### 2.5.4 Assessment of overlap

To assess the extent of citation overlap among primary studies included in the systematic reviews, a citation matrix was constructed, and the Corrected Coverage Area (CCA) index was employed to quantify the overall degree of overlap. The CCA is calculated as follows (12):


CCA=(N−r)/(r×c−r)100%

where N denotes the total number of records identified across all included systematic reviews, r denotes the number of unique primary studies, and c denotes the number of included systematic reviews. Interpretation of CCA values followed the empirically derived thresholds proposed by Pieper et al. (13): < 5% (slight), 5–10% (moderate), 11–15% (High), and > 15%(very high).

#### 2.5.5 Integrated evidence synthesis and clinical relevance

In addition to presenting review-level effect estimates, we performed an outcome-level descriptive synthesis to facilitate cross-review interpretation. For each prespecified outcome, we summarized the number of contributing reviews, the proportion reporting statistically significant effects, the range of review-level sample sizes, the distribution of GRADE certainty ratings, and the AMSTAR 2 methodological ratings. Because the same primary RCT could appear in more than one review, review-level sample sizes were not summed. To distinguish statistical significance from potential clinical importance, we also compared raw-scale effect estimates and their 95% CIs, where interpretable, with published minimal clinically important difference/minimal important change (MCID/MIC) values for stroke populations (14–16). This MCID/MIC comparison was *post hoc* and descriptive: thresholds vary by stroke phase, baseline function, and anchor, and standardized effect sizes cannot be directly compared with raw-scale MCID/MIC values. The comparison was therefore used only to contextualize the magnitude and precision of reported effects and did not alter GRADE ratings.

## 3 Results

### 3.1 Literature search findings

Chinese and English databases were searched, and 405 potentially relevant articles were identified. After duplicates were removed with EndNote X9, 224 remained. Two reviewers independently screened titles and abstracts by using pre-specified criteria, selecting 52 for full-text review. Critical appraisal of full texts yielded 19 eligible studies included in this review (17–35). Further details are presented in Figure 1.

**Figure 1:**
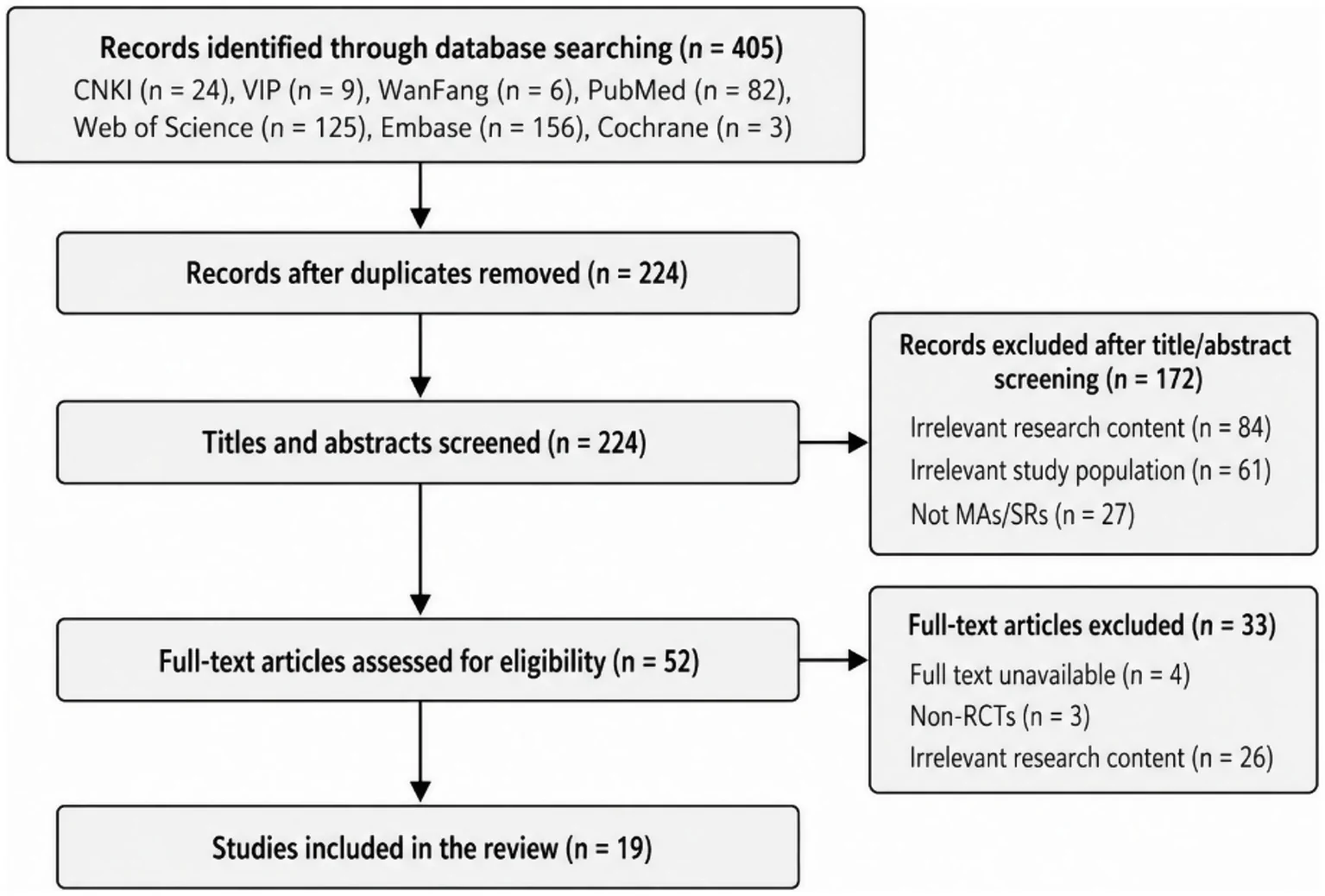
Flowchart of literature screening. MAs, meta-analyses; SRs, systematic reviews; RCTs, randomized controlled trials.

### 3.2 Basic characteristics of included studies

The key characteristics of the 19 included studies are summarized in Table 1, 2. Among them, eight studies (17–19, 23, 29, 33–35) were published in Chinese and 11 (20–22, 24–28, 30–32) in English, with publication years spanning from 2013 to 2026. Regarding risk-of-bias assessment tools, one study (25) employed the RoB 2.0 tool exclusively, one (28) applied the PEDro and RoB 2.0 tools, four (22, 24, 26, 27) used the PEDro scale only, and the remaining 13 studies (17–21, 23, 29–35) relied solely on the Cochrane Risk-of-Bias Tool.

**Table 1:** Summary of basic characteristics of included studies (*n* = 19).

Literature/year of publication	Country region	Study design	Number of included publications	Sample Size	Intervention (EG)	Comparison (CG)	Outcome measures	Conclusion	Quality assessment tools
Luo et al. (2023) (23)	China	MA	22	902	RAGT+CT	CT	①②④⑥	(+)	Cochrane Handbook
Gao et al. (2026) (35)	China	MA	22	998	RAGT	CT	①②③④	(+)	Cochrane Handbook 5.1.0
Guo et al. (2013) (17)	China	SR	12	498	RAGT	CT	①②	(+)	Cochrane Handbook
Li et al. (2018) (18)	China	MA	5	219	RAGT	CT	①②	(+)	Cochrane Handbook
Chang et al. (2024) (29)	China	MA	22	865	RAGT+CT	CT	①②③⑤	(+)	Cochrane Handbook 5.1.0
Peng et al. (2025) (34)	China	MA	9	467	RAGT+CT	CT	①②	(+)	Cochrane Handbook
Guo et al. (2025) (33)	China	MA	13	731	RAGT+CT	CT	①②③	(+)	Cochrane Handbook 5.1.0
Yang et al. (2018) (19)	China	MA	12	642	RAGT+CT	CT	①②③	(+)	Cochrane Handbook 5.1.1
Chen et al. (2024) (24)	China	SR/MA	27	1,167	RAGT+CT	CT	①③④	(+)	PEDro
Hao et al. (2025) (30)	China	SR/MA	41	3,279	RAGT+CT	CT	①②③④	(+)	Cochrane Handbook
Hu et al. (2024) (25)	China	SR/MA	24	1,103	RAGT+CT	CT	①②③④⑤⑥	(+)	Cochrane Handbook 2.0
Lee et al. (2024) (26)	South Korea	SR/MA	19	756	RAGT+CT	CT	①②③④⑤⑥	(+)	PEDro
Liang et al. (2024) (27)	China	SR/MA	28	1,251	RAGT+CT	CT	①②③④⑥	(+)	PEDro
Liu et al. (2025) (31)	China	SR/MA	6	213	FEAT+CT	CT	①③④⑥	(+)	Cochrane Handbook
Moucheboeuf et al. (2020) (21)	France	MA	33	1,466	RAGT +CT	CT	①③⑤	(+)	Cochrane Handbook
Wang et al. (2025) (32)	China	SR/MA	12	651	RAGT+CT	CT	①②④⑤	(+)	Cochrane Handbook
Wu et al. (2023) (22)	China	SR/MA	21	709	RATG+CT	CT	①②③⑤	(+)	PEDro
Zheng et al. (2019) (20)	China	SR/MA	31	1,249	RAGT	CT	①⑤	(+)	Cochrane Handbook
Yang et al. (2024) (28)	China	SR/MA	34	1,166	RAGT	CT	①②③④⑤	(+)	PEDro、Cochrane Handbook 2.0

EG, experimental group; CG, control group; MA, meta-analysis; SR, systematic review; RAGT, robot-assisted gait training; CT, conventional (i.e., standard or routine) rehabilitation training. For outcome measures, “+” indicates a statistically significant difference between the experimental and control groups, whereas “−” indicates no statistically significant difference. Abbreviations for functional assessment tools are as follows: (1) BBS, Berg Balance Scale; (2) FMA, Fugl–Meyer Assessment; (3) FAC, Functional Ambulation Category; (4) 6MWT, 6-Minute Walk Test; (5) TUG, Timed Up and Go Test; and (6) 10MWT, 10-Meter Walk Test. Cochrane Handbook: Cochrane Handbook for Systematic Reviews of Interventions.

**Table 2:** Technology scope of the included systematic reviews.

Review	Technology category	Scope / examples when explicitly specified
Luo et al. (2023) (23)	Mixed RAGT systems	Not restricted to a single device family; pooled different robot-assisted gait-training platforms.
Gao et al. (2026) (35)	Multiple RAGT modalities	Network meta-analysis of different robot-assisted gait-training interventions; heterogeneous platforms.
Guo et al. (2013) (17)	Mixed lower-limb rehabilitation robots	Not restricted to a single robotic platform.
Li et al. (2018) (18)	Mixed lower-limb rehabilitation robots	Early rehabilitation; not restricted to one device family.
Chang et al. (2024) (29)	Exoskeleton-specific	Rehabilitation exoskeleton robots.
Peng et al. (2025) (34)	Exoskeleton-specific	Lower-limb exoskeleton robots.
Guo et al. (2025) (33)	Mixed lower-limb rehabilitation robots	Not restricted to a single robotic platform.
Yang et al. (2018) (19)	Mixed lower-limb rehabilitation robots	Not restricted to a single robotic platform.
Chen et al. (2024) (24)	Mixed RAGT systems	Multiple robot-assisted gait-training systems; not restricted to one device family.
Hao et al. (2025) (30)	Exoskeleton + end-effector	Both major lower-limb robot categories were included; device-type subgrouping was limited by available data.
Hu et al. (2024) (25)	Exoskeleton + end-effector	Examples included Lokomat, BEAR-H1 and HAL (exoskeletons) and Gait Trainer, Gait Master and Morning Walk (end-effectors).
Lee et al. (2024) (26)	Overground exoskeleton	Overground robotic-exoskeleton gait training.
Liang et al. (2024) (27)	Mixed RAGT systems	Robot-assisted gait-training platforms; not restricted to a single device family.
Liu et al. (2025) (31)	Flexible/soft exoskeleton	Flexible exoskeleton-assisted lower-limb training.
Moucheboeuf et al. (2020) (21)	Exoskeleton + end-effector	Examples included Lokomat, Ekso, HAL and H2 (exoskeletons) and GT Trainer (end-effector).
Wang et al. (2025) (32)	Exoskeleton + end-effector	Included both categories and reported subgroup analyses by robot type.
Wu et al. (2023) (22)	Lokomat exoskeleton	Lokomat robotic-orthosis system.
Zheng et al. (2019)(20)	Mixed robot-assisted therapy	Balance-focused review; not restricted to one robotic platform.
Yang et al. (2024) (28)	Robotic exoskeleton	Exoskeleton-specific review of lower-limb function, activity, and participation.

The classification reflects each review’s stated intervention scope. “Mixed” indicates that the review was not restricted to a single device family. The table is intended to make technological heterogeneity explicit and does not imply equivalence among device types.

### 3.3 Methodological quality assessment results of included studies

AMSTAR 2 assessment revealed several methodological limitations across the 19 included systematic reviews. Specifically, funding sources for the primary studies were unreported in 19 reviews (17–35); pre-registration of the review protocol prior to conduct was absent in seven reviews (17–19, 23, 29, 34, 35); and a comprehensive search strategy, including all relevant databases, search terms, and date ranges, was not employed in three reviews (18, 19, 34). A partial application of a comprehensive search strategy was observed in nine reviews (17, 22, 24, 27, 28, 31–33, 35), whereas 14 reviews (17, 20, 21, 23, 25–32, 34, 35) provided only partial lists of excluded studies, and one review (19) omitted such a list entirely. With regard to study characterization, six reviews (18, 19, 21, 23, 29, 35) offered only partial descriptive detail on included studies, two (24, 25) provided no detailed description, and two (33, 34) failed to integrate risk-of-bias assessments into their interpretation of findings. Furthermore, five reviews (19, 20, 22, 23, 26) inadequately addressed observed statistical or clinical heterogeneity, and another five (17, 19, 28, 31, 32) neither assessed publication bias nor discussed its potential influence on the robustness of conclusions. Seven reviews (17–19, 23, 30, 33, 34) disclosed no information regarding potential conflicts of interest. Overall, quality appraisal yielded three high-quality, six moderate-quality, six low-quality, and four very low-quality reviews. Further details are presented in Table 3.

**Table 3:** AMSTAR 2 assessment results for included studies.

Literature/year of publication	Q1	Q2*	Q3	Q4*	Q5	Q6	Q7*	Q8	Q9*	Q10	Q11*	Q12	Q13*	Q14	Q15*	Q16	Quality grade
Luo et al. (2023) (23)	Y	N	Y	Y	Y	Y	PY	PY	Y	N	Y	Y	Y	N	Y	N	L
Gao et al. (2026) (35)	Y	N	Y	PY	Y	Y	PY	PY	Y	N	Y	Y	Y	Y	Y	Y	L
Guo et al. (2013) (17)	Y	N	Y	PY	Y	Y	PY	Y	Y	N	Y	Y	Y	Y	N	N	CL
Li et al. (2018) (18)	Y	N	Y	N	Y	Y	Y	PY	Y	N	Y	Y	Y	Y	Y	N	CL
Chang et al. (2024) (29)	Y	N	Y	Y	Y	Y	PY	PY	Y	N	Y	Y	Y	Y	Y	Y	L
Peng et al. (2025) (34)	Y	N	Y	N	Y	Y	PY	Y	Y	N	Y	Y	N	Y	Y	N	CL
Guo et al. (2025) (33)	Y	Y	Y	PY	Y	Y	Y	Y	Y	N	Y	Y	N	Y	Y	N	L
Yang et al. (2018) (19)	Y	N	Y	N	Y	Y	N	PY	Y	N	Y	N	Y	N	N	N	CL
Chen et al. (2024) (24)	Y	Y	Y	PY	Y	Y	Y	N	Y	N	Y	Y	Y	Y	Y	Y	M
Hao et al. (2025) (30)	Y	Y	Y	Y	Y	Y	PY	Y	Y	N	Y	Y	Y	Y	Y	N	M
Hu et al. (2024) (25)	Y	Y	Y	Y	Y	Y	PY	N	Y	N	Y	Y	Y	Y	Y	Y	M
Lee et al. (2024) (26)	Y	Y	Y	Y	Y	Y	PY	Y	Y	N	Y	Y	Y	N	Y	Y	M
Liang et al. (2024) (27)	Y	Y	Y	PY	Y	Y	PY	Y	Y	N	Y	Y	Y	Y	Y	Y	H
Liu et al. (2025) (31)	Y	Y	Y	PY	Y	Y	PY	Y	Y	N	Y	Y	Y	Y	N	Y	L
Moucheboeuf et al. (2020) (21)	Y	Y	Y	Y	Y	Y	PY	PY	Y	N	Y	Y	Y	Y	Y	Y	H
Wang et al. (2025) (32)	Y	Y	Y	PY	Y	Y	PY	Y	Y	N	Y	Y	Y	Y	N	Y	L
Wu et al. (2023) (22)	Y	Y	Y	PY	Y	Y	Y	Y	Y	N	Y	Y	Y	N	Y	Y	M
Zheng et al. (2019) (20)	Y	Y	Y	Y	Y	Y	PY	Y	Y	Y	Y	Y	Y	N	Y	Y	H
Yang et al. (2024) (28)	Y	Y	Y	PY	Y	Y	PY	Y	Y	N	Y	Y	Y	Y	N	Y	M

Y, Yes; PY, Partially applicable; N, No; *, Key item (s). H, High quality; M, Moderate quality; L, Low quality; CL, Critically low quality.

### 3.4 Evaluation results of the reporting completeness of included studies

The mean PRISMA 2020 checklist score was 19.1 out of 27 (range: 14.5–23.5). Among the 19 included studies, one article (21) scored below 15 points, 16 articles (17–20, 22–28, 31–35) scored between 15 and 20 points, and two articles (29, 30) scored between 21 and 27 points. No study achieved the maximum possible score of 27 points. According to the PRISMA statement guidelines, reporting for any item was deemed substantially incomplete if the proportion of studies adequately addressing that item fell below 50%. The most frequently underreported items were as follows: item 23 (Discussion; 47.4% completeness), item 19 (Individual Study Results; 42.1%), item 2 (Abstract; 31.6%), item 24 (Registration and Protocol; 31.6%), and item 10 (Data Items; 25.3%). Items 5 (Eligibility Criteria) and 14 (Assessment of Reporting Bias) demonstrated 5.3% completeness, whereas item 21 (Reporting Bias) was not reported in any of the included studies (0% completeness). All remaining 19 items were reported with ≥ 50% completeness, indicating satisfactory adherence to those reporting standards.

### 3.5 Evaluation results of the evidence level of included studies

This study conducted a systematic literature search and incorporated 19 peer-reviewed articles. A total of 70 outcome measures were extracted, and the quality of evidence for each measure was rigorously assessed in accordance with the GRADE framework. The evaluated outcome measures included the BBS, FMA-LE, TUG, 6MWT, 10MWT, and FAC. The GRADE assessment results indicated that evidence supporting 20 outcome measures was rated as moderate quality, 32 as low quality, and 18 as very low quality. Detailed classifications are presented in Table 4.

**Table 4:** GRADE assessment results.

Literature	Outcomes					
	BBS	FMA-LE	FAC	6MWT	TUG	10MWT
Luo et al. (2023) (23)	Low	Low	-	Moderate	-	Moderate
Gao et al. (2026) (35)	Very low	Moderate	Very low	Moderate	-	-
Guo et al. (2013) (17)	Very low	Moderate	-	-	-	-
Li et al. (2018) (18)	Low	Moderate	-	-	-	-
Chang et al. (2024) (29)	Very low	Moderate	Low	-	Moderate	-
Peng et al. (2025) (34)	Low	Low	-	-	-	-
Guo et al. (2025) (33)	Low	Low	Moderate	-	-	-
Yang et al. (2018) (19)	Low	Very low	Very low	-	-	-
Chen et al. (2024) (24)	Low	-	Low	Moderate	-	-
Hao et al. (2025) (30)	Low	Very low	Moderate	Low	-	-
Hu et al. (2024) (25)	Moderate	Very low	Low	Moderate	Moderate	Low
Lee et al. (2024) (26)	Low	Low	Low	Very low	Low	Low
Liang et al. (2024) (27)	Low	Low	Low	Low	-	Low
Liu et al. (2025) (31)	Low	-	Low	Moderate	-	Very low
Moucheboeuf et al. (2020) (21)	Very low	-	Low	-	Very low	-
Wang et al. (2025) (32)	Moderate	Moderate	0	Low	Low	-
Wu et al. (2023) (22)	Moderate	Low	Moderate	-	Very low	-
Zheng et al. (2019) (20)	Very low	-	-	-	Low	-
Yang et al. (2024) (28)	Very low	Low	Very low	Very low	Very low	-

BBS, Berg Balance Scale; FMA-LE, Fugl–Meyer Assessment-Lower Extremity subscale; FAC, Functional Ambulation Category; 6MWT, 6-Minute Walk Test; TUG, Timed Up and Go Test; 10MWT, 10-Meter Walk Test. “—” denotes that this outcome measure has not been reported in the existing literature.

### 3.6 Overlap among original studies included in the SR/MAs

The 19 reviews contained 393 primary-study occurrences corresponding to 168 unique primary studies. The overall CCA was 7.44%, indicating moderate overlap. Leave-one-review-out analyses showed that the overall overlap estimate was not materially driven by any single review.

### 3.7 Results of primary outcome assessments

#### 3.7.1 Balance function

A total of 19 studies (17–35) investigated the effect of robotic interventions on BBS scores among stroke survivors with lower-limb motor impairment, as shown in Table 5. According to the GRADE methodology, the overall quality of the evidence was rated as moderate, based on an assessment of three included studies. A meta-analysis conducted by Hu et al. (25) demonstrated a substantial and statistically significant increase in mean BBS scores following robotic intervention [mean difference, MD = 4.55, 95% CI (3.00, 6.11), *p* < 0.001]. Similarly, Wang et al. (32) found that lower-limb robotic rehabilitation yielded significantly greater improvements in balance than conventional therapy [MD = 6.98, 95% CI (3.06, 10.89), *p* < 0.001]. Furthermore, Wu et al. (22) reported that robot-assisted training conferred superior balance-enhancing effects relative to traditional rehabilitation approaches [MD = 2.71, 95% CI (1.39, 4.03), *p* < 0.001].

**Table 5:** Effect size of robot-assisted therapy on BBS in patients with stroke.

Literature	Effect size	95% CI	*p*-value	Statistical significance
Luo et al. (2023) (23)	3.29^a^	(1.64,4.94)	<0.001	Significant
Gao et al. (2026) (35)	3.59^a^	(0.25,6.93)	0.04	Significant
Guo et al. (2013) (17)	3.13^a^	(0.80,5.45)	0.008	Significant
Li et al. (2018) (18)	0.62^b^	(0.32,0.92)	<0.001	Significant
Chang et al. (2024) (29)	3.62^a^	(1.21,6.03)	0.003	Significant
Peng et al. (2025) (34)	2.24^c^	(0.57,3,91)	0.009	Significant
Guo et al. (2025) (33)	6.40^a^	(5.74,7.05)	<0.001	Significant
Yang et al. (2018) (19)	7.20^a^	(4.18,10.23)	<0.0001	Significant
Chen et al. (2024) (24)	-0.55^b^	(−0.99,-0.12)	0.01	Significant
Hao et al. (2025) (30)	10.64^a^	(8.04,13.25)	<0.001	Significant
Hu et al. (2024) (25)	4.55^a^	(3.00,6.11)	<0.001	Significant
Lee et al. (2024) (26)	0.05^a^	(−0.23,0.32)	0.74	Non-significant
Liang et al. (2024) (27)	1.18^b^	(−0.16,3.37)	0.071	Non-significant
Liu et al. (2025) (31)	3.21^a^	(0.96,5.46)	0.005	Significant
Moucheboeuf et al. (2020) (21)	4.16 ^a^	(2.60,5.71)	0.000	Significant
Wang et al. (2025) (32)	6.98^a^	(3.06,10.89)	<0.001	Significant
Wu et al. (2023) (22)	2.71^a^	(1.39,4.03)	<0.001	Significant
Zheng et al. (2019) (20)	4.64^a^	(3.22,6.04)	<0.01	Significant
Yang et al. (2024) (28)	2.34^a^	(0.21,4.47)	0.03	Significant

^a^Mean difference, ^b^standardized mean difference, ^c^weighted mean differences. BBS, the Berg Balance Scale.

#### 3.7.2 Motor function of lower limbs

A total of 15 studies (17–19, 22, 23, 25–30, 32–35) investigated the efficacy of robot-assisted rehabilitation training on lower-limb motor function, as measured by the FMA-LE scale, in stroke survivors with post-stroke lower-limb motor impairment, as shown in Table 6. According to the GRADE evidence quality grading standard, the evidence level was ranked from high to low, and the recently published high-quality research results were preferentially presented. Specifically, Gao et al. (35) reported a mean difference [MD = 4.02, 95% CI (3.51–4.53), *p* < 0.001] in favor of the robot-assisted group relative to controls. Chang et al. (18) found a standardized mean difference [SMD = 2.63,95% CI (1.87,3.38), *p* < 0.001], indicating superior effects of robot-assisted training during early-phase rehabilitation. Similarly, Wang et al. (32) demonstrated significant gains in motor function, including reflex integrity and intermuscular coordination [MD = 3.17,95% Cl (2.34,3.99), *p* < 0.001].

**Table 6:** Effect size of robot-assisted therapy on FMA-LE in patients with stroke.

Literature	Effect Size	95% CI	*p*-value	Statistical significance
Luo et al. (2023) (23)	3.04^a^	(1.73,4.35)	<0.001	Significant
Gao et al. (2026) (35)	4.02^a^	(3.51,4.53)	<0.001	Significant
Guo et al. (2013) (17)	3.97 ^a^	(2.84,5.10)	<0.001	Significant
Li et al. (2018) (18)	0.76^b^	(0.46,1.07)	<0.001	Significant
Chang et al. (2024) (29)	2.63^a^	(1.87,3.38)	<0.001	Significant
Peng et al. (2025) (34)	2.30^c^	(1.39,3.21)	<0.001	Significant
Guo et al. (2025) (33)	3.87^a^	(2.87,4.87)	<0.001	Significant
Yang et al. (2018) (19)	4.69^a^	(2.40,6.97)	<0.001	Significant
Hao et al. (2025) (30)	5.05^a^	(3.90,6.20)	<0.001	Significant
Hu et al. (2024) (25)	2.10^a^	(0.62,3.59)	0.005	Significant
Lee et al. (2024) (26)	0.21^a^	(−0.04,0.47)	0.12	Non-significant
Liang et al. (2024) (27)	0.32^b^	(0.10,0.55)	0.005	Significant
Wang et al. (2025) (32)	3.17^a^	(2.34,3.99)	<0.001	Significant
Wu et al. (2023) (22)	1.27^a^	(−0.88,3.42)	0.25	Non-significant
Yang et al. (2024) (28)	1.15^a^	(0.29,2.01)	0.009	Significant

^a^Mean difference, ^b^standardized mean difference, ^c^weighted mean differences. FMA-LE, Fugl-Meyer Assessment—Lower Extremity.

#### 3.7.3 Walking capacity

Thirteen studies (19, 21, 22, 24–31, 33, 35) investigated the effect of robotic interventions on FAC scores in stroke survivors with lower-limb motor impairment, as shown in Table 7. According to the GRADE evidence quality grading standard, the evidence level was ranked from high to low. Specifically, Guo et al. (33) observed a significantly higher mean FAC score in the robot-assisted therapy group than in the control group (MD = 0.65, 95% CI (0.50, 0,81), *p* < 0.001]. Similarly, Hao et al. (30) demonstrated that adding robot-assisted training to conventional rehabilitation yielded greater improvement in FAC scores than conventional rehabilitation alone [MD = 0.81, 95% CI (0.48, 1.13), *p* < 0.001]. Conversely, three studies (23, 26, 27, 31) reported that robot-assisted walking training conferred no additional benefit over conventional walking training.

**Table 7:** Effect size of robot-assisted therapy on FAC in patients with stroke.

Literature	Effect Size	95% CI	*p*-value	Statistical significance
Gao et al. (2026) (35)	0.44^a^	(0.02,0.87)	0.04	Significant
Chang et al. (2024) (29)	0.30 ^a^	(−0.01,0.61)	0.06	Non-significant
Guo et al. (2025) (33)	0.65^a^	(0.50,0.81)	<0.001	Significant
Yang et al. (2018) (19)	0.90^a^	(0.46,1.34)	<0.001	Significant
Chen et al. (2024) (24)	0.32^b^	(0.01,0.63)	0.04	Significant
Hao et al. (2025) (30)	0.81^a^	(0.48,1.13)	<0.001	Significant
Hu et al. (2024) (25)	0.44^a^	(0.23,0.65)	<0.001	Significant
Lee et al. (2024) (26)	0.18^a^	(−0.01,0.37)	0.07	Non-significant
Liang et al. (2024) (27)	0.32^b^	(0.10,0.55)	0.005	Significant
Liu et al. (2025) (31)	0.13^a^	(−0.25,0.51)	0.50	Non-significant
Moucheboeuf et al. (2020) (21)	0.51^a^	(0.07,0.95)	0.022	Significant
Wu et al. (2023) (22)	0.28^a^	(0.11,0.45)	0.001	Significant
Yang et al. (2024) (28)	0.25^a^	(0.02,0.48)	0.03	Significant

^a^Mean difference, ^b^standardized mean difference. FAC, Functional Ambulation Category.

#### 3.7.4 Walking endurance

A total of 10 studies (23–28, 30–32, 35) investigated the effect of robotic interventions on 6MWT performance in stroke survivors with lower-limb motor impairment, as shown in Table 8. Among these, seven studies (23–26, 30–32, 35) reported statistically significant improvements in 6MWT distance following robotic therapy, although the overall quality of evidence ranged from moderate to very low. By contrast, three studies (27, 28) found no statistically significant difference in 6MWT outcomes between robotic intervention and control groups; both were rated as providing low or very low quality evidence. Specifically, Luo et al. (23) observed a significantly greater improvement in walking endurance in the robotic intervention group than in the control group [MD = 0.05 95% CI (0.01, 0.1), *p* = 0.02]. Similarly, Gao et al. (35) reported a significantly larger gain in 6MWT distance in the robotic group versus controls [MD = 20.62, 95% CI: 12.71–28.53, *p* < 0.001].

**Table 8:** Effect size of robot-assisted therapy on 6MWT in patients with stroke.

Literature	Effect Size	95% CI	*p*-value	Statistical significance
Luo et al. (2023) (23)	0.05^a^	(0.01,0.10)	0.02	Significant
Gao et al. (2026) (35)	20.62^a^	(12.71,28.53)	<0.001	Significant
Chen et al. (2024) (24)	25.14^b^	(10.19,40.09)	0.001	Significant
Hao et al. (2025) (30)	63.98^a^	(35.50,92.45)	<0.001	Significant
Hu et al. (2024) (25)	30.66^a^	(22.36,38.97)	<0.001	Significant
Lee et al. (2024) (26)	0.11^a^	(−0.07,0.28)	0.23	Non-significant
Liang et al. (2024) (27)	0.11^b^	(−0.10,0.32)	0.30	Non-significant
Liu et al. (2025) (31)	51.97^a^	(34.66,69.27)	<0.001	Significant
Wang et al. (2025) (32)	16.02^a^	(6.67,25.37)	<0.001	Significant
Yang et al. (2024) (28)	1.65^a^	(−13.36.16.66)	0.083	Non-significant

^a^Mean difference, ^b^standardized mean difference. 6MWT, 6-Minute Walk Test.

#### 3.7.5 Functional mobility

Eight studies (20–22, 25, 26, 28, 29, 32) investigated the effect of robotic interventions on the TUG test performance among stroke survivors with lower-limb motor impairment, as shown in Table 9. Among these, two studies (20, 21, 25, 26, 29) reported statistically significant improvements in standing and ambulation function following robotic therapy; however, the overall quality of evidence for these findings ranged from moderate to very low. Conversely, six studies (22, 28, 32) found no statistically significant effect of robotic interventions on TUG outcomes or related functional mobility measures, with corresponding evidence quality rated as low to very low. According to the GRADE evidence quality grading standard, the evidence level was ranked from high to low. Specifically, Chang et al. (29) demonstrated that exoskeleton-based robotic rehabilitation yielded a significantly greater reduction in TUG time than conventional therapy [MD = −2.77, 95% CI = (−4.48 to −1.05), *p* = 0.002]. Similarly, Hu et al. (25) reported a substantial and statistically robust improvement in TUG performance with robotic assistance [MD = −4.05, 95% CI (−5.12, −2.98), *p* < 0.001].

**Table 9:** Effect size of robot-assisted therapy on TUG in patients with stroke.

Literature	Effect size	95% CI	*p*-value	Statistical significance
Chang et al. (2024) (29)	-2.77^a^	(−4.48, −1.05)	0.002	Significant
Hu et al. (2024) (25)	−4.05^a^	(−5.12, −2.98)	<0.001	Significant
Lee et al. (2024) (26)	−0.15^a^	(−0.45,0.15)	0.34	Non-significant
Moucheboeuf et al. (2020) (21)	3.20^a^	(−2.58,8.98)	0.277	Non-significant
Wang et al. (2025) (32)	−1.37_a_	(−6.88,4.14)	0.63	Non-significant
Wu et al. (2023) (22)	−0.12^a^	(−0.71,0.46)	0.68	Non-significant
Yang et al. (2024) (28)	−0.01^a^	(−1.59,1.57)	0.99	Non-significant
Zheng et al. (2019) (20)	−5.60^a^	(−13.69,2.49)	0.18	Non-significant

^a^Mean difference, ^b^Timed Up and Go Test.

#### 3.7.6 Gait speed

Five studies (23, 25–27, 31) investigated the effect of robotic-assisted therapy on 10MWT performance in stroke survivors with lower-limb motor impairment, as shown in Table 10. Among these, four studies (23, 27, 31) reported statistically significant improvements in gait speed following robotic intervention, although the overall quality of evidence ranged from moderate to very low. By contrast, Hu et al. (25, 26) found no statistically significant difference in gait speed between robotic-assisted and control interventions, with both reporting low-quality evidence. Notably, Luo et al. (23) demonstrated that robotic-assisted training yielded a modest but statistically significant improvement in gait speed relative to conventional rehabilitation [MD = 0.05, 95% CI (0.01, 0.10), *p* = 0.02].

**Table 10:** Effect size of robot-assisted therapy on 10MWT in patients with stroke.

Literature	Effect size	95% CI	*p*-value	Statistical significance
Luo et al. (2023) (23)	0.05^a^	(0.01,0.1)	0.02	Significant
Hu et al. (2024) (25)	0.06^a^	(−0.01,0.14)	0.06^a^	Non-significant
Lee et al. (2024) (26)	0.28^a^	(0.05,0.51)	0.02	Significant
Liang et al. (2024) (27)	0.1^b^	(0.06,0.14)	<0.01	Significant
Liu et al. (2025) (31)	0.15^a^	(0.04,0.27)	0.01	Significant

^a^Mean difference, ^b^standardized mean difference. 10MWT, 10-Meter Walk Test.

### 3.8 Integrated comparative synthesis across outcomes

Table 11 summarizes cross-outcome consistency, GRADE certainty, AMSTAR 2 quality, and clinical relevance. BBS and FMA-LE showed the most consistent favorable findings; FAC and 6MWT were less consistent, TUG was mainly non-significant, and 10MWT was favorable in 4/5 reviews but mostly supported by low-certainty evidence. Because primary studies overlap, review counts reflect concordance rather than independent replications.

**Table 11:** Integrated outcome-level synthesis of the umbrella evidence.

Outcome	Significant reviews	GRADE (M/L/VL)	AMSTAR 2 (H/M/L/CL)	Brief interpretation
BBS	17/19	3/10/6	3/6/6/4	Consistent benefit; most evidence low/very low. Clinical importance varied (MCID ~6.5–12.5 points) (33).
FMA-LE	13/15	5/7/3	1/5/5/4	Direction was mostly favorable. MIC/MCID is phase-dependent (~3.3–4.3 p Mostly favorable); MIC/MCID is phase-dependent (~3.3–4.3 acute; ~6 chronic) (34, 35).
FAC	10/13	3/7/3	2/6/4/1	Generally favorable but inconsistent and mainly low certainty; a universal stroke-specific MCID is not established.
6MWT	7/10	5/3/2	1/5/40	Mixed; some estimates reached reported MCIDs (~34–71 m), whereas others did not (37).
TUG	2/8	2/3/3	2/4/2/0	Mostly non-significant; additional benefit remains uncertain.
10MWT	4/5	1/3/1	1/2/ 2/0	Often statistically favorable, but mostly low certainty; clinical importance varied (~0.16 m/s benchmark) (36).

M/L/VL, moderate/low/very low GRADE certainty; H/M/L/CL, high/moderate/low/critically low AMSTAR 2 quality. Counts indicate review-level concordance, not independent replications, because primary RCTs overlap. MCID/MIC comparisons are contextual and phase-dependent.

## 4 Discussion

### 4.1 Integrated clinical interpretation: consistency, clinical relevance, and evidence certainty

The cross-outcome synthesis shows the most consistent favorable evidence for BBS (17/19 reviews) and FMA-LE (13/15), although most supporting estimates were low or very low certainty. Walking outcomes were less consistent: FAC and 6MWT showed mixed benefit, TUG was predominantly non-significant, and 10MWT was favorable in four of five reviews but mostly low certainty. Robot-assisted training may therefore improve balance and lower-limb motor impairment as an adjunct to conventional rehabilitation, whereas effects on walking independence, endurance, mobility, and gait speed remain less certain.

The dispersion of review-level effect estimates also requires cautious interpretation. For BBS, for example, Hao et al. (30) reported an effect estimate of 10.64, whereas Lee et al. (26) reported 0.05. These values should not be treated as directly comparable replications, because the reviews differed in patient selection and stroke phase, robotic device class (including mixed lower-limb robotic systems versus overground exoskeletons), training dose, comparator intensity, and the composition of included primary trials. Differences in baseline severity and analytic specifications may also contribute to between-review variation. Accordingly, the magnitude of benefit should be interpreted within the clinical and methodological context of each review rather than from the numerical range alone.

Statistical significance did not consistently imply clinical importance. Reported benchmarks include BBS MCIDs of ~6.5–12.5 points in acute stroke (14), FMA-LE MIC/MCID values of ~3.3–4.3 points in acute stroke and ~6 points in chronic stroke (15, 36), 6MWT MCIDs of ~34–71 m in selected post-stroke subgroups (16), and a gait-speed change of ~0.16 m/s in subacute stroke (37). Some review-level mean differences reached these values, whereas others did not; standardized effects could not be directly mapped to raw-scale thresholds, and confidence intervals sometimes encompassed clinically small effects. Interpretation should therefore consider stroke phase, baseline function, metric, and precision rather than *p* values alone.

Generalizability is further limited by technological and service heterogeneity. The included reviews covered mixed RAGT platforms, exoskeletons, Lokomat-type systems, end-effector devices, and flexible systems, with substantial variation in assistance mode, dose, and comparators; current evidence does not establish superiority of any device class. Quality of life, long-term disability, caregiver burden, institutionalization, and sustained participation were not consistently reported, although these outcomes are particularly relevant in older adults with frailty or comorbidity (7). Real-world adoption also depends on cost, infrastructure, staff training, workflow, access, and sustainability, for which current economic and implementation evidence remains limited (38, 39).

### 4.2 Methodological rigor of included studies requires further enhancement

The present umbrella review was reported in accordance with the PRIOR guideline (8), which promotes transparent reporting of the objectives, methods, results, and limitations of overviews of reviews. Within this framework, AMSTAR 2 was employed to evaluate the methodological quality of the 19 included systematic reviews. Critical appraisal revealed several methodological shortcomings: none of the reviews (17–35) reported the funding sources of the primary studies they incorporated; 14 reviews (17, 20, 21, 23, 25–32, 34, 35) provided only a partial list of excluded studies; nine reviews (17, 22, 24, 27, 28, 31–33, 35) implemented only a partially comprehensive search strategy; seven reviews (17–19, 23, 30, 33, 34) failed to disclose any potential conflicts of interest; and seven reviews (17–19, 23, 29, 34, 35) lacked a prespecified, registered protocol prior to conduct. AMSTAR 2 scoring indicated that three reviews were rated as high quality, six as moderate quality, six as low quality, and four as critically low quality, yielding an overall moderate methodological quality across the corpus. Future systematic reviews and meta-analyses should adhere strictly to the PRISMA reporting guidelines; explicitly document funding sources for all included primary studies; comprehensively declare all potential conflicts of interest; fully report the search strategy; and prospectively register a detailed, peer-reviewed protocol prior to initiation to enhance rigor and transparency.

### 4.3 Reporting completeness of the included studies require enhancement

In this study, the mean PRISMA adherence score was 19.1 (range: 14.5–23.5), reflecting a moderate overall quality of reporting and suggesting several methodological shortcomings in the current literature. Key deficiencies identified included the following: (i) failure to assess the risk of bias arising from missing outcome data (i.e., reporting bias); (ii) absence of a detailed description of the methodology employed to evaluate such bias during data synthesis; (iii) lack of explicit criteria or rationale for grouping studies prior to synthesis; (iv) omission of a comprehensive list and definition of all prespecified variables for data extraction, including interventions, comparators, outcomes, and sources of funding; (v) absence of protocol registration or a prospectively documented review plan; and (vi) incompleteness of the abstract with respect to essential reporting elements. The following are recommended to enhance methodological rigor and transparency in future systematic reviews: prospectively registering study protocols, explicitly describing procedures for study grouping and synthesis, and systematically evaluating and reporting on risks of bias related to missing or selectively reported outcomes.

### 4.4 Overall evidence certainty was generally low

Across the 70 outcome estimates evaluated with GRADE, none was rated high certainty; 20 (28.6%) were moderate, 32 (45.7%) low, and 18 (25.7%) very low. Thus, 71.4% of the available outcome evidence was low or very low certainty. The principal reasons for downgrading were risk of bias, inconsistency from unexplained clinical and statistical heterogeneity, imprecision from small samples or wide confidence intervals, and potential publication bias. This distribution reinforces the outcome-specific interpretation above: a statistically significant estimate supported by low- or very-low-certainty evidence should not be interpreted as equally reliable as a moderate-certainty estimate, and no outcome in this umbrella review was supported uniformly by high- or moderate-certainty evidence. Accordingly, the conclusions of this umbrella review remain constrained by the moderate-to-low methodological quality of the underlying reviews and should be interpreted as conditional rather than definitive estimates of clinical benefit.

### 4.5 Study limitations

This study followed established systematic review standards and comprehensively evaluated robotic interventions for lower-limb motor recovery after stroke. Key limitations include: (1) only English- and Chinese-language full-text publications were included, which may have introduced language bias; (2) the reliability of this reappraisal depends on the methodological quality of the 19 included reviews, many of which were rated low or critically low by AMSTAR 2; (3)Moderate primary-study overlap (CCA = 7.44%) reduces the independence of review-level findings, so counts of reviews and significant results should not be treated as cumulative independent evidence.; (4) Heterogeneity in device type, training dose, rehabilitation phase, comparators, and outcome definitions limited direct and device-specific inference; (5) the MCID/MIC comparison was a *post hoc* descriptive analysis, and available thresholds vary by stroke phase, baseline function, and anchor; (6) quality of life, long-term disability, caregiver burden, institutionalization, participation, and health-economic outcomes were not consistently reported by the included reviews and therefore could not be synthesized as outcomes of this umbrella review; and (7) implementation and economic studies discussed for clinical context were not part of the prespecified umbrella-review search. Restricting inclusion to English- and Chinese-language reviews may also have introduced language bias and excluded relevant evidence from other regions, thereby limiting the global generalizability of the findings.

### 4.6 Future research directions

Future research should prioritize three areas:(1) Conduct adequately powered, prospectively registered RCTs with transparent allocation methods and consistent reporting of adverse events, adherence, attrition, and longer-term outcomes; evidence syntheses should follow PRISMA/PRIOR and assess outcome-specific certainty.(2) Standardize reporting of robot type, assistance mode, treatment dose, co-interventions, stroke stage, and baseline walking ability, and use prespecified subgroup or comparative analyses to clarify which patients and device categories benefit most.(3) Use a core outcome set spanning motor impairment, walking, activities of daily living, participation, quality of life, falls, and sustained community mobility. Report 95% CIs with phase-appropriate MCID/MIC benchmarks where available, and include implementation and economic outcomes such as staff requirements, throughput, acceptability, equitable access, and cost-effectiveness.

These priorities would help clarify not only whether robot-assisted training is effective, but also which patients benefit most, which devices and treatment doses are appropriate, and whether early gains lead to sustained functional recovery.

## 5 Conclusion

Current evidence is most directionally consistent for balance and lower-extremity motor impairment, but the magnitude of benefit is not uniformly clinically important and no outcome is supported by high-certainty evidence. Findings for walking independence, endurance, functional mobility, and gait speed are more inconsistent. Moderate overlap among reviews, substantial technological heterogeneity, and predominantly low- or very-low-certainty evidence limit claims of independent replication and generalizability. Robot-assisted training should therefore be considered an adjunct whose value depends on patient goals, device characteristics, and local implementation context. Future research should integrate clinically meaningful thresholds with patient-centered, long-term, implementation, and economic outcomes.

## Data Availability

The original contributions presented in the study are included in the article/Supplementary material, further inquiries can be directed to the corresponding authors.
