# Supplementary material for: Robot-assisted training for post-stroke lower-limb dysfunction: an umbrella review
Source: Front Public Health. 2026 Oct 16;14:1969488. doi: 10.3389/fpubh.2026.1969488 (PMC13719321; doi:10.3389/fpubh.2026.1969488)
Supplement: Supplementary file 1 [file Table_1.DOCX]

**The full search strategy**

**CNKI**(**Chinese)：**

（机器人 + 外骨骼 + 康复机器人 + 机器人辅助）AND（卒中 + 缺血性卒中 + 脑梗死 + TIA + 脑出血 + 中风 + 脑血管意外）AND（下肢运动功能 + 下肢 + 行走 + 步态 + 下肢运动 + 平衡）AND（系统评价 + 荟萃分析 + Meta分析）

(Robot + exoskeleton + rehabilitation robot + robot assistance) AND (stroke + ischemic stroke + cerebral infarction + TIA + cerebral hemorrhage + stroke + cerebrovascular accident) AND (lower limb movement function + lower limb + walking + gait + lower limb movement + balance) AND (systematic review + meta-analysis + meta-analysis)

(Note: The English versions are translations provided for transparency and that the searches were executed using the original Chinese terms with syntax adapted to each database.)

**Wanfang Data** (**Chinese)：**

（机器人 OR 外骨骼 OR 康复机器人 OR 机器人辅助）AND（卒中 OR 缺血性卒中 OR 脑梗死 OR TIA OR 脑出血 OR 中风 OR 脑血管意外）AND（下肢运动功能 OR 下肢 OR 行走 OR 步态 OR 下肢运动 OR 平衡）AND（系统评价 OR 荟萃分析 OR Meta分析）

(Robot OR exoskeleton OR rehabilitation robot OR robot-assisted) AND (stroke OR ischemic stroke OR cerebral infarction OR TIA OR cerebral hemorrhage OR stroke OR cerebrovascular accident) AND (lower limb movement function OR lower limb OR walking OR gait OR lower limb movement OR balance) AND (systematic review OR meta-analysis OR meta-comparison)

(Note: The English versions are translations provided for transparency and that the searches were executed using the original Chinese terms with syntax adapted to each database.)

**VIP Chinese Science and Technology Periodical Database (chinese):**

（机器人 OR 外骨骼 OR 康复机器人 OR 机器人辅助）AND（卒中 OR 缺血性卒中 OR 脑梗死 OR TIA OR 脑出血 OR 中风 OR 脑血管意外）AND（下肢运动功能 OR 下肢 OR 行走 OR 步态 OR 下肢运动 OR 平衡）AND（系统评价 OR 荟萃分析 OR Meta分析）

(Robot OR exoskeleton OR rehabilitation robot OR robot-assisted) AND (stroke OR ischemic stroke OR cerebral infarction OR TIA OR cerebral hemorrhage OR stroke OR cerebrovascular accident) AND (lower limb movement function OR lower limb OR walking OR gait OR lower limb movement OR balance) AND (systematic review OR meta-analysis OR meta-comparison)

(Note: The English versions are translations provided for transparency and that the searches were executed using the original Chinese terms with syntax adapted to each database.)

**PubMed:**

((((((("Robotics"[Mesh]) OR (robotics[Title/Abstract])) OR (robotic[Title/Abstract])) OR (robot[Title/Abstract])) OR (exoskeleton[Title/Abstract])) AND ((((((((((((((((((("Stroke"[Mesh]) OR (cerebrovascular disorders[Title/Abstract])) OR (stroke[Title/Abstract])) OR (cerebrovascular event[Title/Abstract])) OR (poststroke[Title/Abstract])) OR (apoplexy[Title/Abstract])) OR (cerebrovascular accident[Title/Abstract])) OR (brain vascular accident[Title/Abstract])) OR (CVD[Title/Abstract])) OR (cerebral infarction[Title/Abstract])) OR (brain infarction[Title/Abstract])) OR (brain ischemia[Title/Abstract])) OR (intracranial embolism[Title/Abstract])) OR (cerebral hemorrhage[Title/Abstract])) OR (cerebral haemorrhage[Title/Abstract])) OR (brain hemorrhage[Title/Abstract])) OR (brain haemorrhage[Title/Abstract])) OR (intracranial hemorrhage[Title/Abstract])) OR (intracranial haemorrhage[Title/Abstract]))) AND (((((((("Lower Extremity"[Mesh]) OR (Lower limb motor function[Title/Abstract])) OR (lower extremity[Title/Abstract])) OR (walking[Title/Abstract])) OR (walk[Title/Abstract])) OR (balance[Title/Abstract])) OR (gait[Title/Abstract])) OR (lower limb[Title/Abstract]))) AND ((("Meta-Analysis" [Publication Type]) OR (meta-analysis[Title/Abstract])) OR (systematic reviews[Title/Abstract]))

**Cochrane Library：**

#1 MeSH doscriptor: [Robotics] explode all trees

#2 ("robotics" OR "robotic" OR "robot" OR "exoskeleton"):ti,ab,kw

#3 #1 OR #2

#4 MeSH descriptor: [Stroke] explode all trees

#5（"cerebrovascular disorders" OR "stroke" OR "cerebrovascular event" OR "poststroke" OR "apoplexy‘ OR 'cerebrovascular accident" OR "brain vascular accident" OR “CVD" OR "cerebral infarction” OR “brain infarction” OR “brain ischemia” OR “intracranial embolism” OR “cerebral hemorrhage” OR “cerebral haemorrhage” OR “brain hemorrhage” OR “brain haemorrhage” OR “intracranial hemorrhage” OR “intracranial haemorrhage”）

#6 #4 OR #5

#7 MeSH descriptor: [Lower Extremity] explode all trees

#8 ("Lower limb motor function" OR "lower extremity" OR "walking" OR "walk" OR "balance" OR "gait" OR "lower limb")t.ab,kw

#9 #7 OR #8

#10 MeSH descriptor: [Meta-Analysis as Topic] explode all trees

#11 (systematic reviews):ti,ab,kw

#12 #10 OR #11

#13 #3 AND #6 AND #9

**Embase：**

#1 'robotics'/exp OR 'robotics':ti,ab OR 'robotic':ti,ab OR 'robot':ti,ab OR 'exoskeleton':ti,ab

#2 'stroke'/exp OR 'cerebrovascular disorders':ti,ab OR 'stroke':ti,ab OR 'cerebrovascular event':ti,ab OR 'poststroke':ti,ab OR 'apoplexy':ti,ab OR 'cerebrovascular accident':ti,ab OR 'brain vascular accident':ti,ab OR 'cvd':ti,ab OR 'cerebral infarction':ti,ab OR 'brain infarction':ti,ab OR 'brain ischemia':ti,ab OR 'intracranial embolism':ti,ab OR 'cerebral hemorrhage':ti,ab OR 'cerebral haemorrhage':ti,ab OR 'brain hemorrhage':ti,ab OR 'brain haemorrhage':ti,ab OR 'intracranial hemorrhage':ti,ab OR 'intracranial haemorrhage':ti,ab)) AND

#3 'lower extremity'/exp OR 'lower limb motor function':ti,ab OR 'lower extremity':ti,ab OR 'walking':ti,ab OR 'walk':ti,ab OR 'balance':ti,ab OR 'gait':ti,ab OR 'lower limb':ti,ab

#4 'meta analysis'/exp OR 'meta-analysis':ti,ab OR 'systematic reviews':ti,ab

#5 #1 AND #2 AND #3 AND #4

**Web of Science：**

"robotics" OR "robotic" OR "robot" OR "exoskeleton" AND "cerebrovascular disorders" OR "stroke" OR "cerebrovascular event" OR "poststroke" OR "apoplexy‘ OR 'cerebrovascular accident" OR "brain vascular accident" OR “CVD" OR "cerebral infarction” OR “brain infarction” OR “brain ischemia” OR “intracranial embolism” OR “cerebral hemorrhage” OR “cerebral haemorrhage” OR “brain hemorrhage” OR “brain haemorrhage” OR “intracranial hemorrhage” OR “intracranial haemorrhage” AND "Lower limb motor function" OR "lower extremity" OR "walking" OR "walk" OR "balance" OR "gait" OR "lower limb" AND “meta-analysis” OR “systematic reviews”
